# Neurological Short-Term Outcomes of a Cohort of Children Born to Zika Virus-Infected Mothers in Barcelona

**DOI:** 10.3390/children9101537

**Published:** 2022-10-09

**Authors:** Natàlia Romaní, Maria Pieras, Marie Antoinette Frick, Elena Sulleiro, Carlota Rodó, Aroa Silgado, Anna Suy, Maria Espiau, Claire Thorne, Carlo Giaquinto, Ana Felipe-Rucián, Pere Soler-Palacín, Antoni Soriano-Arandes

**Affiliations:** 1 Pediatric Infectious Diseases and Immunodeficiencies Unit, Vall d’Hebron Research Institute, Hospital Universitari Vall d’Hebron, Universitat Autònoma de Barcelona, 08035 Barcelona, Spain; 2 Centro de Investigación Biomédica en Red de Enfermedades Infecciosas, Department of Microbiology, Instituto de Salud Carlos III, Hospital Universitari Vall d’Hebron, 08035 Barcelona, Spain; 3 Maternal Fetal Medicine Unit, Department of Obstetrics, Hospital Universitari Vall d’Hebron, Vall d’Hebron Barcelona Hospital Campus, Passeig Vall d’Hebron 119-129, 08035 Barcelona, Spain; 4 Population, Policy and Practice Research and Teaching Department, Great Ormond Street Institute of Child Health, University College London, London WC1N 1EH, UK; 5 Division of Paediatric Infectious Diseases, Department of Women’s and Children’s Health, University-Hospital of Padua, Via Giustiniani 3, 35128 Padua, Italy; 6 Pediatric Neurology Section, Pediatric Neurology Research Group, Vall d’Hebron Research Institute, Hospital Universitari Vall d’Hebron, Universitat Autònoma de Barcelona, 08035 Barcelona, Spain

**Keywords:** Zika virus, Zika virus infection, arboviruses, microcephaly, congenital infection, adverse outcome, travel-associated, children, neonate, mother-to-child transmission

## Abstract

Zika virus (ZIKV) is a vector-borne flavivirus with a known teratogenic effect, yet the full spectrum has not been delineated. Studies on endemic areas tried to characterize the clinical outcomes of ZIKV intrauterine exposure. We aimed to describe early neurodevelopmental outcomes on prenatally ZIKV-exposed children in a non-endemic ZIKV area. This is a prospective observational cohort study conducted from May 2016 to December 2021 at Hospital Universitari Vall d’Hebron in Barcelona, Catalonia, Spain. We monitored for up to 24 months 152 children extracted from a pregnant women cohort with suspected ZIKV infection; eleven women (11/150; 7.3%) fulfilled the criteria for a confirmed ZIKV infection. Among the 152 children included, we describe two cases of congenital ZIKV syndrome (CZS) born from women with a confirmed ZIKV infection. Additionally, we describe five cases of other potentially ZIKV-related outcomes (OPZROs), all with normal birth cranial circumference and born to women with probable ZIKV infection. The low exposed prevalence of adverse outcomes in asymptomatic children at birth in a non-endemic area suggests that close follow-up should be addressed by primary care pediatricians instead of pediatric specialists. Further studies are needed to assess the effects of ZIKV intrauterine exposure beyond two years of life.

## 1. Introduction

Zika virus (ZIKV) is mainly a vector-borne flavivirus related to epidemic outbreaks worldwide, especially in Latin American countries [1]. However, ZIKV has already been associated with other transmission routes such as sexual intercourse, maternal–fetal transmission, or blood transfusion. Mother-to-child transmission is the main concern for pediatricians. In fact, the association of ZIKV infection during pregnancy with adverse fetal outcomes [2,3] led the World Health Organization (WHO) to declare ZIKV as a Public Health Emergency of International Concern (PHEIC) in February 2016 [4].

Congenital ZIKV syndrome (CZS) severity and prognosis are directly associated with the head circumference (HC) at birth and central nervous system (CNS) damage. However, apparently healthy and asymptomatic infants at birth may eventually develop abnormalities detected in CNS imaging or in subsequent neurodevelopment assessments [5,6]. Few children with intrauterine exposure to ZIKV and normocephalic at birth have been proven to present abnormal neurologic development [7]. Because of the uncertainty regarding the long-term neurologic health of newborns exposed to ZIKV in utero, the Centers for Disease Control and Prevention recommended neurodevelopmental assessment and follow-up of these newborns through early childhood [8].

In addition to the limited knowledge about the long-term effects of ZIKV intrauterine infection on neurocognitive development, published studies have been conducted in areas with active vector-borne transmission of ZIKV [6,9,10,11]. We aimed to describe the early neurodevelopmental outcomes of a cohort of prenatally exposed ZIKV infants born in a non-endemic vector-borne ZIKV area, focusing on those children with normal prenatal imagining and asymptomatic at birth.

## 2. Materials and Methods

This is a uncenter prospective observational cohort study of children born to ZIKV-infected mothers at the Hospital Universitari Vall d’Hebron in Barcelona, Catalonia, Spain, between May 2016 to December 2021.

We used REDCap^®^ (Vanderbilt University, Nashville, TN, US) for data collection of the cohort of the children. Ethical approval was obtained from the committee of the center with the number PR(AMI)103/2016, and all the parents signed an informed consent before the inclusion of their babies in the study, and the study protocol followed the Spanish consensus document for ZIKV infection [8].

### 2.1. Study Population

A total of 152 newborns born to 150 mothers with confirmed or probable ZIKV infection during pregnancy (after travelling or coming from a ZIKV vector-borne endemic area) was included.

#### 2.1.1. Mother’s Cohort

##### Enrollment Criteria

All the pregnant women testing positive for ZIKV-IgG or ZIKV-IgM and (i) travelling to endemic areas during the pregnancy or the two previous months, or (ii) who had sexual partners that had visited endemic areas in the last 6 months were eligible for recruitment.

We collected demographic data, blood, and/or urine samples to test for ZIKV, and the samples were collected and analyzed with reverse transcriptase polymerase chain reaction (RT-PCR) (RealStar^®^Zika Virus RT-PCR kit 1.0, Altona Diagnostics), ZIKV-IgG, and ZIKV-IgM (IIFT Arboviral fever Mosaic IgG and IgM, Euroimmun, Germany from 2016 to December 2017; and ELISA Virus Zika IgG and IgM, Euroimmun, Germany, from December 2017 to the end of study) (Table 1). In cases where ZIKV RT-PCR tested negative but ZIKV-IgG was positive, samples were shipped to the national reference laboratory Instituto de Salud Carlos III (ISCIII) located in Madrid. They processed the samples using a specific plaque reduction neutralization test for ZIKV (ZIKV-PRNT). Unfortunately, ISCIII did not perform PRNT for other flaviviruses; therefore, positive ZIKV-PRNT titers were not able to be compared with other flaviviruses. A neutralization titer ≥1/32 was considered positive, detecting the presence of ZIKV-neutralizing antibodies. The ZIKV test date was considered to be the diagnosis date for ZIKV infection [12].

##### Case Definition

Pregnant women were classified at risk to be exposed to ZIKV regardless of the symptoms. Table 1 shows the classification of the laboratory ZIKV diagnosis for pregnant women [13]. The ZIKV-PRNT test was not available during the COVID-19 pandemic. Consequently, and in order to include all possible ZIKV infections during the pregnancy, a small subgroup of ZIKV IgG-positive mothers with unknown ZIKV-PRNT results were also recruited.

#### 2.1.2. Children’s Cohort

##### Enrollment Criteria

The resulting newborns of the maternal cohort were recruited at birth and were followed up for a maximum period of 2 years.

At recruitment, we collected demographic data, including birth date, gender, country of birth, and parents’ demographic data, especially the mother’s country of birth. In addition to all this data, laboratory tests (blood and urinary test) and microbiological analysis (RT-PCR in serum, urine, cerebrospinal fluid (CSF), and other samples such as saliva) were performed on the majority of the newborns. Serology confirmation in children was carried out at birth and during follow-up until negativization. Unfortunately, we could not perform ZIKV-PRNT in those newborns with positive ZIKV-IgG due to the lack of infrastructure in our reference laboratory.

##### Case Definition

A confirmed congenital ZIKV infection was defined by microbiological analysis: (i) positive ZIKV RT-PCR in serum, urine, saliva, amniotic fluid, or CSF; and/or (ii) positive ZIKV IgM; and/or (iii) positive ZIKV IgG beyond 18 months old, or by compatible neuroimaging or phenotypic characteristics consistent with CZS.

### 2.2. Cohort Follow-Up and Endpoints

We created a monographic agenda dedicated exclusively to follow-up and to assess these children in our center. During this time, visits were scheduled at 1, 4, 9, 12, 18, and 24 months of age. On each visit, we collected anthropological data (weight, length, and head circumference); physical examination and neurodevelopment assessments (thorough neurological exam and Ages & Stages Questionnaires Third Edition (ASQ-3)); serological analysis were tested until seroreversion; and at least one of ophthalmological check-up with an ocular fundus, hearing evaluation (Auditory Brainstem Response), and radiological imaging (cranial ultrasound or magnetic resonance imaging (MRI)) were performed.

Loss of follow-up was recorded, and the reason for that loss was registered in the known cases.

### 2.3. Statistical Analysis

We used Stata^®^ 15 (StataCorp) for statistical analyses. To analyze the association between maternal and infant characteristics regarding confirmed or probable maternal infection status, we used χ2 or Fisher exact tests and reported *p*-values for each test.

## 3. Results

Between May 2016 and December 2021, 152 children were included that were extracted from a cohort of 150 pregnant women. Referrals were based on maternal laboratory assay results suggestive of ZIKV infection during pregnancy.

### 3.1. Characteristics of the Participants in the Cohort

Eleven women (11/150; 7.3%) had a confirmed ZIKV infection, 7/144 were positive for blood ZIKV RT-PCR, 2/17 for amniotic fluid ZIKV RT-PCR, 1/94 for placenta ZIKV RT-PCR, and 7/150 (4.6%) were positive for ZIKV-IgM alongside a positive ZIKV-PRNT. The remaining 92.7% were probable ZIKV infections, 126/150 (84%) were positive for ZIKV-PRNT, and 13/150 (8.6%) had unknown ZIKV-PRNT status.

Most of the women were immigrants or visiting friends and relatives coming from Latin American countries (95.7%), whereas European-born women travelling for tourism to Latin America represented less than 5% of the cohort (7/163; 4.7%). Of the whole group, 119/152 (79.3%) were asymptomatic.

Among the 152 children included in the study, 70 (46.7%) were male and 12/152 (7.9%) were preterm (<37 weeks’ gestation). The mean (SD) gestational age was 39 (38–40) weeks, and the median (IQR) birth weight was 3240 g (2930–3515) with a median (IQR) z-score of −0.39 (−1 to 0.21). The median (IQR) birth cranial circumference was 34 cm (33–35) with a median (IQR) z-score of −0.35 (−1.1 to 0.38). The results of the mother–child pairs national cohort, with the main newborn outcomes just after delivery, have been previously published [12].

Among 11 mothers with a confirmed ZIKV infection, we described two cases of CZS. The remaining nine children were asymptomatic at birth; four presented normal neurological development during follow-up, and five children were lost on follow-up between 4 and 9 months of life. Among women classified as a probable ZIKV infection during pregnancy, we described five cases of OPZROs: a perinatal stroke and four cases of neurodevelopmental impairment detected during follow-up. All of them were asymptomatic and had a within-range cranial circumference at birth.

### 3.2. Adherence to Follow-Up

**Figure 1:**
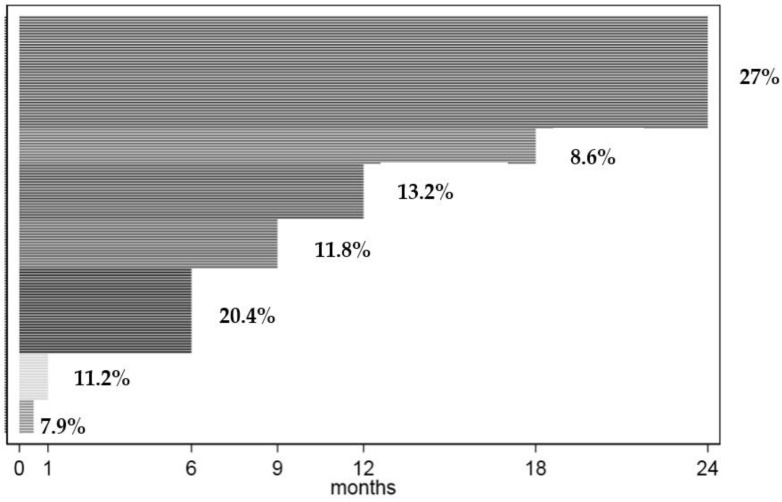
Individual follow-up time. Percentage (%) of children followed up to 1, 6, 12, 18, and 24 months.

The total follow-up time was 1829.00 months (24.50 in those two confirmed congenital ZIKV infection, and 1804.5 in the rest of the children), with a median follow-up time of 9.00 (12.25 for confirmed congenital ZIKV infection vs. 9.00 for the rest of the children) months; thus, 48.8% of the patients were followed up to 12 months, and only 27% completed the 24-month follow-up (Figure 1). There was a high follow-up loss rate. Most families either returned to their home country during the COVID-19 pandemic or were unaware of the need for follow-up of their otherwise healthy baby.

**Figure 2:**
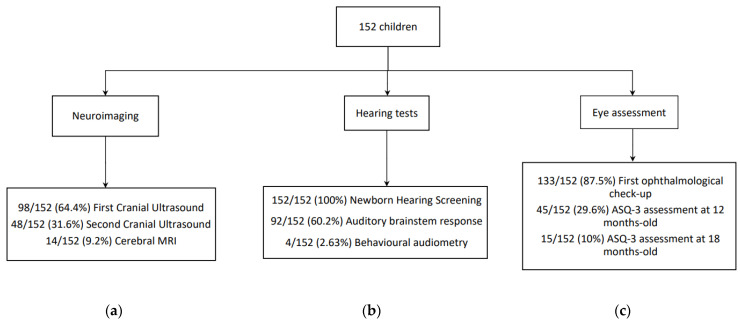
Children (number (percentage, %)) who underwent the assessment tests during follow-up. (**a**) Proportion (percentage) of children who underwent the neuroimaging tests. (**b**) Proportion (percentage) of children who underwent hearing tests. (**c**) Proportion (percentage) of children whose ASQ-3 assessment was collected and whose first ophthalmological check-up was done.

Consequently, the collected data during follow-up is shown in the figure (Figure 2).

Among these 152 children, we here showed the two cases of ZIKV congenital infection. No mother–infant pairs in the cohort were diagnosed as having coinfections. The rest of the participants were defined as children with probable ZIKV intrauterine exposure (150/152; 98%).

### 3.3. Description of ZIKV Congenital Infection

We here describe two congenital infections caused by ZIKV and five cases of other potentially ZIKV-related outcomes (OPZROs).

#### 3.3.1. ZIKV Congenital Infection

##### Case 1

We describe a case of medical abortion in the second term of pregnancy. This woman did not recall any symptoms related to a ZIKV infection but tested positive for serum ZIKV RT-PCR, ZIKV-IgM, ZIKV-IgG, and ZIKV-PRNT at 15 weeks of pregnancy. Cerebral and musculoskeletal malformations were detected on the ultrasound at 20 weeks’ gestation. A fetal MRI was performed showing incipient microcephaly with cerebral atrophy and bilateral ventriculomegaly as well as cerebellar hypoplasia. In a posterior fetal ultrasound, generalized edema and club feet were also detected. Given these findings, obstetricians performed an amniocentesis, and ZIKV RT-PCR in amniotic fluid tested positive. Medical abortion was indicated at 22 weeks of pregnancy, and samples were obtained from the fetus testing positive for ZIKV RT-PCR in serum, CSF, and placenta as well as positive for ZIKV-IgM and ZIKV-IgG. The fetal autopsy confirmed the presence of microcephaly, cerebral atrophy, and ventriculomegaly with frequent parenchymal calcifications, bilateral polymicrogyria, and glioneuronal heterotopia. In the pathological anatomy of the placenta, particles morphologically compatible with viruses were found in the chorionic villi.

##### Case 2

Secondly, we present a case of CZS with positive ZIKV RT-PCR in amniotic fluid and ZIKV-IgG but negative serum ZIKV RT-PCR and ZIKV-IgM at birth. It was a symptomatic infection in the first trimester of the pregnancy (9–10 weeks of pregnancy). The mother reported having a non-pruriginous, maculopapular rash the day she returned from Colombia that lasted 3 days. At 11 weeks of pregnancy, the serum tested positive for ZIKV RT-PCR and ZIKV-IgG with an initially negative ZIKV-IgM. Posterior blood samples showed a positive ZIKV-IgM at 21 weeks of pregnancy and negative again from the 26th week of pregnancy. ZIKV RT-PCR was negative from the 21st week of pregnancy. Furthermore, the initial study also tested positive for Dengue virus (DENV) IgM and IgG. At 19 weeks of pregnancy, prenatal echography abnormalities such as microcephaly and intrauterine growth retardation were detected. Amniocentesis was performed during the second trimester with a positive ZIKV RT-PCR and a negative DENV RT-PCR in amniotic fluid. Further imaging studies were conducted such as a fetal MRI at the 2nd and 3rd trimesters that were consistent with echogenic findings. Genetic studies performed with the amniotic fluid ruled out any fetal chromosomopathy.

Postnatal MRI showed severe microcephaly (−4SD), parenchymal calcifications, ventriculomegaly, lissencephaly, and pachygyria. Physical examination at birth highlighted increased muscle tone (hypertonia) with vivid osteotendinous reflexes (hyperreflexia). At birth, she presented with abnormal visual and auditory brainstem responses that normalized at 6 months old. The ocular fundus showed focal pigmentary retinal mottling affecting the macula at birth that softened over time, and ophthalmological monitoring showed persistent esotropia and hypertropia.

During the neurodevelopment follow-up, she developed a profound intellectual disability, spastic tetraparesis, epilepsy, dysphagia, and failure to thrive. Intermittent treatment with botulism toxin for spastic tetraparesis was started at 20 months of age. The liquid dysphagia was addressed with thickening agents. Lastly, the failure to thrive was treated successfully with nutritional supplements. She had the first epileptic seizure at the age of 2 years old in the context of a respiratory infection, and then antiepileptic treatment was initiated.

Periodic blood tests were done since birth showing persistent positive ZIKV IgG with seroreversion between 12 and 20 months old.

#### 3.3.2. Other Potential ZIKV-Related Outcomes (OPZROs)

##### Case 3. Perinatal Stroke

Among the studied children, one presented tonic–clonic seizures early at birth with neuroimaging compatible with an embolic stroke. The cranial circumference at birth was normal (percentile 11). His mother tested positive for ZIKV-IgG and ZIKV-PRNT but negative for ZIKV-IgM and RT-PCR during the pregnancy. At 12 h of life, the patient presented with frequent hemiclonal seizures that required more than two antiepileptics to control the seizures. A cerebral MRI was consistent with a perinatal stroke, predominantly on the left posterior middle cerebral artery territory and bilateral border territory of the middle cerebral artery and posterior cerebral artery. All microbiological tests at birth were negative, including CSF samples for ZIKV RT-PCR, ZIKV-IgM, and ZIKV-IgG. ZIKV-IgM and IgG in serum were not done at birth but were negative at 4 months old. We had no other subsequent microbiological studies. Cardiological tests ruled out a cardiac cause of stroke. Neurologic development during follow-up (language use, motor and social skills) was appropriate with no appreciable impairment on the right side and normal ASQ-3 tests.

##### Case 4. Mild Neurodevelopmental Disorder

Case 4 was child with probable ZIKV intrauterine exposure with a positive ZIKV-IgG and negative serum for ZIKV RT-PCR and ZIKV-IgM at birth. Seroreversion was determined at 4 months old with a negative ZIKV-IgG. The cranial circumference at birth was normal (percentile 68). During follow-up, we detected delayed language acquisition with some autistic spectrum features. Hearing tests and cranial ultrasound at birth were normal. Microarray Comparative Genomic Hybridization (array CGH) was performed and interpreted as normal. A posterior cerebral MRI showed craniofacial disproportion suggestive of microcephaly with stable cranial circumference growth (+1SD). During follow-up, he improved progressively, persisting with a slight delay in language use and short attention span at 2 years old.

##### Case 5. Global Neurodevelopmental Delay

Case 5 was a child born to an asymptomatic mother who was returning from Honduras during the first trimester of the pregnancy. After arrival, her mother tested positive for ZIKV-IgG and ZIKV-PRNT. At birth, she was positive for ZIKV-IgG with negative ZIKV RT-PCR and IgM. Seroreversion was determined at 12 months of age with negative ZIKV-IgG. She first presented with proper neurodevelopment with correct audiometric and visual tests. The cranial circumference at birth was normal (60th percentile). However, at 12 months old, the physical examination showed slight axial hypotonia. In addition, the ASQ-3 highlighted an impairment in gross and fine motor spheres and altered social interaction. She was monitored by neuropediatricians that determined a global developmental delay with a severe delay in language acquisition and social interaction. Axial hypotonia and marked hyperlaxity in the lower limbs persisted in physical examination over time. Furthermore, we observed progressive microcephaly from the 48th percentile at 12 months old to the 11th percentile at 24 months old. Further studies such as a cerebral MRI and a metabolopathy screening showed no other alterations.

##### Case 6. Specific Language Impairment (SLI)

Case 6 was a child with a positive ZIKV-IgG at birth whose mother presented positive ZIKV-IgG and ZIKV-PRNT during the pregnancy. Seroreversion of the baby was determined at 10 months old. Visual tests and neuroimaging were normal at birth. The cranial circumference at birth was normal (78th percentile). The auditory brainstem response showed mixed hearing loss with posterior normal behavioral audiometry. However, at 12 months old, a delay in language acquisition was detected, and the ASQ-3 showed impairment in social interaction. He was referred to a neuropediatrician who confirmed a specific language disorder with attention deficit hyperactivity disorder (ADHD) features. A cerebral MRI at 15 months old was normal. He improved progressively during follow-up and was discharged at 2 years old.

##### Case 7. Mild Neurodevelopment Disorder

Lastly, case 7 was a child with probable ZIKV intrauterine exposure. In this case, the newborn tested positive for ZIKV-IgG with a negative ZIKV-IgM and serum ZIKV RT-PCR. Seroreversion was determined at 7 months old. Visual and neuroimaging at birth were normal. The cranial circumference at birth was normal (14th percentile). However, auditory brainstem response showed mild hearing loss that resolved in subsequent controls. At 12 months old, the ASQ-3 test was abnormal in the problem-solving sphere. During follow-up, we observed a delay in language acquisition with difficulties in social interaction and social conduct and a short attention span. He improved over time and was discharged at 2 years old.

#### 3.3.3. Neuroimaging Findings Not Attributed to ZIKV Exposure

Twelve children presented nonspecific and various abnormal findings on cranial ultrasounds with no clinical repercussions. Regarding cerebral MRI findings, 2/8 showed signs of microcephaly, 2/8 highlighted the presence of germinolytic cysts with no other abnormalities, one patient had findings related to prematurity, and finally one presented with nonspecific cerebral white matter impairment. These children with cerebral MRI findings presented normal neurologic development during follow-up.

## 4. Discussion

In this cohort, we describe cases of early neurological impairment in children prenatally exposed to ZIKV in non-endemic areas. This cohort was extracted from a maternal cohort based on serological results when returning from an endemic area (mostly visiting friends and relatives or emigrating to Spain). A specific schedule was created to assess these children appropriately for up to 24 months.

We present two cases of confirmed CZS: one medical abortion at 22 weeks of pregnancy due to antenatal malformations, and a live-born infant with clinical and microbiological findings compatible with ZIKV congenital infection. Both mothers had confirmed ZIKV infections during pregnancy. Additionally, both children tested positive for ZIKV RT-PCR in amniotic fluid, but only the medical abortion tested positive for RT-PCR in serum and ZIKV-IgM. These findings agree with Rodó et al. [14], who suggested the fetal immune system is able to solve the infection during intrauterine life. In addition, both neuroimaging studies showed microcephaly, parenchymal calcifications, and cerebral atrophy. The second case developed a severe intellectual disability, spastic tetraparesis, dysphagia, and failure to thrive during follow-up.

We also identified five cases of OPZROs, following the phenotype description published by Ades et al. [15], all of them born to mothers with a probable ZIKV infection. One child presented with a perinatal embolic stroke and four with mild neurodevelopmental disorders. Neonatal seizures and stroke are characteristic of vertically transmitted infections. ZIKV is highly neurotropic with an affinity for placental tissue that leads to mother to fetus transmission and neurologic sequelae [16,17]. ZIKV in utero exposure has been associated with seizures and early-life strokes in children [16,18,19]. Additionally, higher rates of autism spectrum disorder (ASD) have been reported in congenital infections, and therefore congenital ZIKV infection has also been studied as a risk factor for ASD [9,20]. Furthermore, Faiçal et al. [10] and Mulkey et al. [11] already described decreased neurodevelopmental scores over time on ZIKV prenatally exposed children without microcephaly at birth. We present a rate of mild neurodevelopmental disorders (4/150 (2.6%)) similar to the prevalence described in our region [21] and other studies on Spanish and worldwide populations [22,23].

Recently more studies expressed the need for further follow-up to evaluate the long-term effects of intrauterine exposure to ZIKV [6,7,9,10,20,24,25,26,27]. They imply that ZIKV can induce a spectrum of subtle and silent injuries to the fetal brain that can appear through childhood and adolescence [17,19,20,24,25,26,27]. Although we cannot assess the relation between our results and ZIKV prenatal exposure, we agree on the need for further study on the long-term effects of ZIKV on the fetal brain.

Compared with American cohort studies [6,9], our cohort has detected a small number of adverse outcomes that may be related to ZIKV infection during pregnancy. One of the potential explanations is that our maternal cohort shortened the time of exposure to ZIKV during pregnancy compared to those that live throughout the pregnancy in a ZIKV-endemic area. Therefore, the possible impact on the fetus of the repeated exposure is not reflected in our cohort and can be considered a limitation of the study. On the other hand, unstandardized maternal screening and different ZIKV intrauterine exposure diagnostic criteria could also explain the discrepancy in the results.

It is also important to note the great challenges that ZIKV laboratory diagnosis has still to resolve due to the short viremia period and the cross-reactivity in serological assays with other flaviviruses. ZIKV-PRNT were not compared to other specific flavoviruses. Additionally, due to the COVID-19 pandemic, ISCIII, our national referral laboratory, did not have the infrastructure to perform ZIKV-PRNT on both children and mothers. Consequently, mother–infant pairs with a low likelihood of ZIKV infection during pregnancy might have been included in the study. At the same time, we could not provide the follow-up data we expected. Many patients were lost on follow-up, as only 27% completed the 24-month follow-up. Therefore, another limitation of our study is that not every child underwent all the tests expected from the study design. Although all of them underwent newborn hearing screening, only 60.2% had a subsequent hearing evaluation. Accordingly, 87.5% had a first ophthalmological check-up, and 64.4% had at least one radiological imaging. Regarding neurological assessment, we only received ASQ-3 results for 10% of the children at 18 months of age. These circumstances impacted our ability to confirm prenatal ZIKV exposure and assess the frequency of OPZROs among exposed children.

## 5. Conclusions

Screening for ZIKV in exposed pregnant women is essential to conduct intensive prenatal ultrasound assessment in infected mothers. In addition, current data on congenital ZIKV infection supports that the spectrum of ZIKV infection in fetuses and infants extends beyond microcephaly and cerebral malformations to a pattern of structural anomalies and functional disabilities. The absence of clinical and radiologic anomalies at birth does not exclude later neurodevelopmental impairment.

In this cohort, we describe 2 cases of CZS among 11 confirmed ZIKV intrauterine exposure. Another 5 children with probable but not confirmed ZIKV exposure presented neurological impairment during follow-up, although the relation between ZIKV exposure and these outcomes is uncertain.

Confirming ZIKV infection in pregnant women and their children is challenging, especially in non-endemic areas, where a short period of ZIKV RNA detection forces the reliance on serologic testing. Health systems need to decide whether to invest time and resources on serological studies or to follow a significant number of children during their early childhood to detect potential neurological abnormalities. However, considering the low prevalence of neurodevelopmental impairment in asymptomatic children at birth in non-endemic areas, close follow-up only by primary care pediatricians seems reasonable.

## Figures and Tables

**Table 1:** Classification of laboratory ZIKV diagnosis for pregnant women and their offspring.

Title 1	RT-PCR	IgG	IgM	ZIKV-PRNT
Confirmed	+			
	− or not performed	+	+	+ ≥1/32
Probable	− or not performed	+	-	+ ≥1/32
	− or not performed	+	+	-
Unlikely ZIKV exposure	− or not performed	+	-	-
	− or not performed	-	-	Not performed

ZIKV: Zika virus; RT-PCR: reverse transcriptase polymerase chain reaction; IgG: immunoglobulin G; IgM: immunoglobulin M; ZIKV-PRNT: plaque reduction neutralization test for ZIKV. During the COVID-19 pandemic, positive ZIKV-IgG and unknown ZIKV-PRNT were also considered a probable ZIKV infection.

## Data Availability

Upon request.
